# Neurogenic Hypertension, the Blood–Brain Barrier, and the Potential Role of Targeted Nanotherapeutics

**DOI:** 10.3390/ijms24032213

**Published:** 2023-01-22

**Authors:** Richard Nii Lante Lamptey, Chengwen Sun, Buddhadev Layek, Jagdish Singh

**Affiliations:** Department of Pharmaceutical Sciences, School of Pharmacy, College of Health Professions, North Dakota State University, Fargo, ND 58105, USA

**Keywords:** Angiotensin, antihypertensive, blood–brain barrier, drug-resistant hypertension, lipid-based nanoparticles, nanotherapeutics, neurogenic hypertension, polymer-based nanoparticles, sympathetic nervous activity

## Abstract

Hypertension is a major health concern globally. Elevated blood pressure, initiated and maintained by the brain, is defined as neurogenic hypertension (NH), which accounts for nearly half of all hypertension cases. A significant increase in angiotensin II-mediated sympathetic nervous system activity within the brain is known to be the key driving force behind NH. Blood pressure control in NH has been demonstrated through intracerebrovascular injection of agents that reduce the sympathetic influence on cardiac functions. However, traditional antihypertensive agents lack effective brain permeation, making NH management extremely challenging. Therefore, developing strategies that allow brain-targeted delivery of antihypertensives at the therapeutic level is crucial. Targeting nanotherapeutics have become popular in delivering therapeutics to hard-to-reach regions of the body, including the brain. Despite the frequent use of nanotherapeutics in other pathological conditions such as cancer, their use in hypertension has received very little attention. This review discusses the underlying pathophysiology and current management strategies for NH, as well as the potential role of targeted therapeutics in improving current treatment strategies.

## 1. Neurogenic Hypertension (NH)

Hypertension is a significant risk factor for renal impairment, other cardiovascular diseases, diabetes mellitus, and multiple end-organ damages [1,2,3]. Although several factors can give rise to hypertension, the primary etiology of most hypertensive cases is unclear. Also, diagnosis of most hypertension cases is often delayed due to their asymptomatic nature [4]. However, insidious changes in cardiovascular function, such as changes in peripheral resistance, stroke volume, cardiac output, baroreceptor sensitivity, and sympathetic nervous system (SNS) output, occur during the development of hypertension [5,6]. The past few decades have seen attention drawn to the impact of the SNS on chronically elevated blood pressure (BP).

Nevertheless, a large pool of recent studies affirms a relatively higher incidence of sympathetic nerve activity (SNA) (as indicated by high levels of norepinephrine and plasma catecholamines) in hypertension unresponsive to conventional treatment, which is also referred to as resistance hypertension or neurogenic hypertension (NH) [7,8]. This strong association between an increase in SNA and elevated BP forms the basis of NH: a form of hypertension mainly driven by a sympathetic mechanism [7,9,10]. Despite identifying this strong association, the exact driver for the increase in SNA is still under investigation. Recent findings suggest that the central action of angiotensin II (Ang II) may be the principal initiator and driving force for increased SNA and subsequent increase in BP [11,12,13,14]. This inference is supported by the increased Ang II levels and activity in the central nervous system (CNS) during hypertension development and progression [9,12,15,16,17,18]. Therefore, therapeutics should be targeted toward the leading cause within the brain to manage NH successfully. However, very few antihypertensive agents are known to enter the brain, and these drugs have severe side effects that have limited their use [19,20,21]. Thus, the biggest challenge for the successful treatment of NH is effectively delivering therapeutic agents to the brain, causing desirable changes to the SNS and significantly reducing BP. This concept of targeted delivery toward managing neurogenic disorders is not novel and has been successfully demonstrated in previously reported publications [22,23,24,25,26]. Although the successful use of targeting nanoparticles is still an area of science under intense investigation, the strides made so far are sufficient to suggest possible development for use in NH. In this review, we explore the pathophysiology of NH, role of the blood–brain barrier (BBB) in limiting the efficacy of conventional antihypertensive therapy, and the prospects of targeted nanoparticles in controlling SNA to achieve BP control.

## 2. Pathophysiology and Proposed Mechanisms of NH

**Figure 1:**
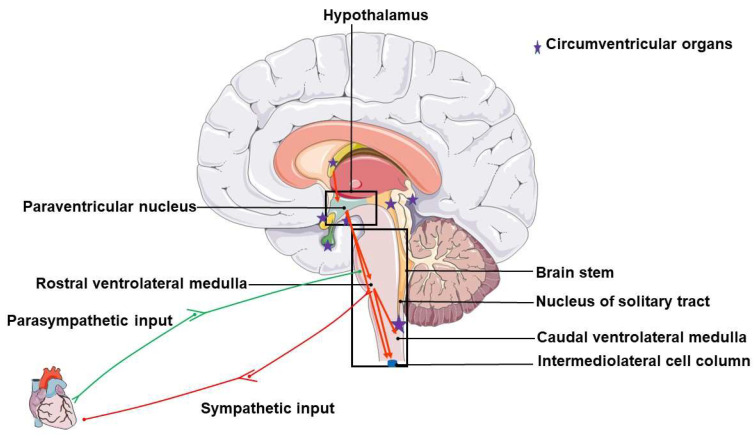
Brain regions that contribute to neurogenic hypertension. Purple stars indicate regions of the circumventricular organs.

Over the past decade, studies have elucidated the exact mechanisms involved in NH’s initiation, development, and progression [1,7,9,16,27,28,29,30]. Major regions within the brain are now known to play critical roles in initiating and maintaining elevated BP in NH through a series of events originating from outside and within the brain. These major regions are highlighted in Figure 1. Below we briefly look at the role played by some brain regions in NH and how they can serve as potential targets for the successful management of NH.

### 2.1. Circumventricular Organs

Like their name, circumventricular organs (CVOs) are a collective term describing structures surrounding the brain’s ventricle, lacking distinct BBB phenotypes, and playing either a secretory or sensory role. [31,32,33,34,35]. In mammals, the CVO includes the subfornical organ (SFO), area postrema (AP), the median eminence (ME), the organum vasculosum of the lamina terminals (OVLT), adjacent neurohypophysis/posterior pituitary (PP), pineal gland, subcommissural organ (SCO), and choroid plexus [36]. Over the years, the role of the CVO in regulating BP has been extensively studied. Ganong and Murakami [37] revealed that CVOs mediate adrenocorticotropic hormone response to peripheral Ang II, promoting thirst and increasing arginine vasopressin levels. In addition, the CVO can permit particular substances to trigger changes in brain function without crossing the BBB. This behavior explains how an increase in peripheral Ang II can cause changes in brain function [38,39]. Since the CVOs lack a distinctive BBB, they appear to be the easiest target for BP control.

### 2.2. The Paraventricular Nucleus (PVN) of the Hypothalamus

The paraventricular nucleus (PVN) is a small essential cluster of neurons within the hypothalamus that functions in development, neuroendocrine roles, growth, regulation of body fluid balance, and gastrointestinal and cardiovascular outputs [40,41,42,43]. Unlike the CVOs, blood vessels in the PVN display characteristic features of BBB, are relatively denser, and undergo structural changes during physiological conditions such as lactation [44,45]. The PVN receives multiple inputs from the CVO via peptidergic neurotransmitters, assesses them, and integrates them to generate complex multifactorial autonomic outputs [40,41,43,46,47]. Although direct projections from the PVN to the spinal cord imply that the PVN can directly influence SNA via glutamatergic input, the PVN also indirectly influences SNA through the pressor region of the RVLM [40,48,49,50]. The PVN glutamatergic neuron stimulation is sufficient to cause autonomic dysfunction which further increases BP, making the PVN a potential target for successful NH management [46,48,49,50,51,52].

### 2.3. Medullary Structures (Nucleus Tractus Solitarius and the Two Ventrolateral Medullas)

The medulla is the lowest part of the brain stem within the brain that connects to the spinal cord and modulates several vital functions of the brain [53]. Although the medulla performs multiple jobs, the primary role of the medulla is regulating cardiovascular functions, such as heart rate and BP [54]. Well-organized structures within the medulla play crucial roles in the reception and transmission of signals in BP regulation. Three distinct regions are the primary sites for baroreflex and cardiovascular integration. 

#### 2.3.1. The Nucleus of the Solitary Tract or Nucleus Tractus Solitarius (NTS)

This is a pair of cell bodies located in the dorsomedial medulla and lateral to the vagus nerve nucleus, which serves as the primary site of cardiorespiratory reflex integration [55,56]. Neurons of the NTS are known to have Ang II-like immunoreactivity, suggesting this neurochemical messenger is vital to their functions [56,57]. The role of the NTS in BP regulation is more of a relay center receiving inputs and transmitting its information to autonomic control regions of the ventrolateral medulla [58]. However, studies point out that inhibition of the NTS functions results in hypertension [59,60,61].

#### 2.3.2. The Caudal Ventrolateral Medulla (CVLM)

This receives inputs from the baroreceptors and transmits them to the RVLM. GABAergic neurons from the caudal ventrolateral medulla provide short-term control of SNA and are vital for maintaining consistent BP [62]. Studies show that CVLM may have anatomical segregations that can promote baroreceptor reflexes or limit SNA [63].

#### 2.3.3. The Rostral Ventrolateral Medulla

This is the central region for the reflex regulation of SNA and resting cardiovascular function [58,61,64]. The RVLM inputs originate from different nuclei within the brain and peripheral nerves. Functional GABA receptors have been found in the RVLM and are associated with its physiological role in regulating BP [65,66,67,68,69]. Inhibition or ablation of the RVLM causes a significant drop in BP, indicating that the RVLM functions to increase or maintain elevated arterial BP and SNA, which is the opposite of the role of the CVLM [58,61,64,70].

## 3. Central Renin–Angiotensin System (RAS) Increases SNA in NH

**Figure 2:**
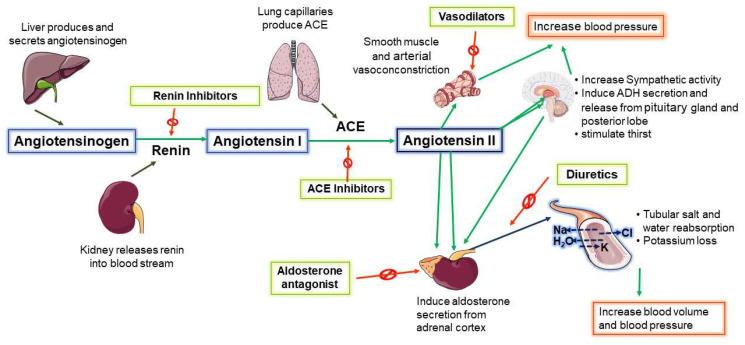
Renin–angiotensin system influence on blood pressure. ACE; Angiotensin Converting Enzyme.

It is an established fact that a higher level of Ang II is associated with NH; however, the source of brain Ang II is a debated subject [71,72]. Nevertheless, Ang II is known to mediate an increase in mean arterial pressure and cardiac sympathetic tone (likely through a unique set of RVLM neurons) while decreasing cardiac parasympathetic tone [16,17,40,73]. Over the past few decades, the role of Ang II in promoting SNA to sustain NH is gradually becoming clear. Under normal conditions, SNS is responsible for the long-term stabilization of BP through heart rate and vascular diameter adjustments with the help of neuroregulators such as epinephrine and norepinephrine [74]. However, sympathetic overactivity initiated by Ang II is believed to be the most likely trigger of chronically elevated BP, which is resistant to conventional antihypertensive drugs (Figure 2) [75].

## 4. Inflammation and Oxidative Stress in NH

Chronic stress significantly contributes to the initiation and progression of NH, a pathophysiology that can be blocked by inhibiting SNA [75,76]. Several studies have revealed the involvement of oxidative stress and proinflammatory molecules (adhesion molecules, such as intercellular adhesion molecules and p-selectin and inflammatory molecules, such as tumor necrosis factor-alpha, C-reactive proteins, interleukin 6 and monocyte chemoattractant protein 1) in NH [29,77]. Higher expressions of these inflammatory markers are observed in the brainstem, NTS, PVN, and RVLM of SHR, indicating that the SHR exhibit a specific inflammatory state responsible for the cardiovascular autonomic dysfunction and development of NH [29,78,79,80]. Also, the number of leukocytes (monocytes and lymphocytes) in SHR is significantly higher than their normotensive controls, the Wistar Kyoto rats (WKY) [81], further affirming the role of inflammation in NH progression.

Inflammatory molecules released from the vascular endothelial cells and leukocytes can enter the brain parenchyma by damaging BBB. Subsequently, these inflammatory molecules activate microglia, leading to increased production of reactive oxygen species (ROS) and chemokines/cytokines, which can directly affect neuronal function [82]. Released chemokines/cytokines from cells within the brain induce sympathoexcitation of the brain neurons. The production of more inflammatory markers from the bone marrow further augments increased sympathoexcitation in the brain. The role of inflammation in the progression of hypertension is also corroborated by several experiments where the blockage of inflammatory pathways resulted in reduced BP in SHR [83,84].

## 5. Current Management Strategies for NH

Effective hypertension management aims to prevent end-organ damage and involves a balance of pharmacologic and nonpharmacologic interventions. Although early detection aids in the management of chronically elevated BP, it is often challenging to have such an ideal situation. The complex nature of NH makes curative treatment difficult. Therefore, the current therapeutic approach to tackling NH aims at bringing BP under control to prevent any cardiovascular events and associated end-organ damage [85]. In addition, surgical procedures that have been explored for NH management interfere with the sympathetic influence on cardiac function. Although several surgical approaches were adopted, only a few progresses toward preclinical stages and still lead to inconclusive outcomes. In the following sections, we will briefly discuss both therapeutic and surgical approaches that have been implemented for the management of NH.

### 5.1. Clinical Management—A Cocktail of Antihypertensive Combination Therapy

The primary consideration for managing NH is to prevent end-organ damage [86,87]. Before drug therapy is initiated, non-pharmacological measures are recommended to reduce BP. Lifestyle modifications such as exercising, reducing alcohol and sodium consumption, and ceasing cigarette smoking improve BP control [88,89]. In addition, some dietary components, such as bamboo shoot extract, have shown significant inhibition of ACE and could have a beneficial effect in lowering BP [90,91]. Monotherapy is the recommended therapeutic approach for managing early-stage hypertension. Yet, the lack of sufficient guidance to clinicians on options for individualizing therapies makes drug choice a significant concern, especially in managing NH. In addition, current knowledge cannot confirm if the available therapeutics for hypertension management can impact central sympathetic outflow associated with NH. 

Despite the abovementioned fact, combination therapies have become the primary practice for controlling elevated BP in NH patients [92,93]. Usually, a combination of three drug classes (and a stepwise addition of a fourth antihypertensive if BP remains elevated) is often prescribed, which include at least a diuretic, an angiotensin receptor blocker (ARB), and/or ACE inhibitor [92]. However, studies of ARBs show they do not reduce sympathetic nervous outflow in NH when administered alone. Even in some cases, they may augment central neural vasoconstrictor outflow with increased plasma norepinephrine levels [94,95]. On the contrary, a study found that adding valsartan (an ARB) to an ACE inhibitor improves cardiac SNA [96]. However, the current treatment guideline does not recommend combining an ARB with an ACE for hypertension management due to the likelihood of causing renal failure [97]. The American heart association makes several recommendations for managing hypertension resistant to monotherapy, including dose augmentation with the addition of beta blockers, hydralazine, and minoxidil. However, most NH patients are elderly and often have associated cardiovascular (e.g., heart failure, cardiac arrhythmia) and non-cardiovascular comorbidities (e.g., diabetes) that can interfere with current treatment choices. Therefore, NH patients should be treated case by case instead of through a generalized treatment regimen [98].

Table 1 provides a list of current antihypertensive drugs that can be used in combination to manage NH.

### 5.2. Centrally Acting Agents

Most of the clinically used BP-reducing drugs function outside the CNS. However, a few antihypertensive agents are known to have central activity, reducing overall vasomotor tone by activating receptors within the ventrolateral medulla. Centrally acting drugs either stimulate imidazoline receptors (rilmenidine, moxonidine) or α-2 (Clonidine) within the central nervous system [101]. Although the central actions of these drugs could be useful in NH, their benefits are marred with several adverse effects that limit their suitability for clinical use. Therefore, dose adjustment is proposed as a strategy to circumvent the harsh side effects of these chemotherapeutics; regardless, this approach has not seen much success. A more promising approach has been the development of second-generation imidazoline binding agents. An example of such an agent is rilmenidine, whose beneficial sympatholytic and BP-lowering effects are augmented by its ability to protect from postural hypotension [102]. In addition to its protective effects, rilmenidine is well tolerated and effective in reducing left ventricular hypertrophy associated with essential hypertension [103,104].

### 5.3. Renal Denervation

In the early 19th century, “Serious hypertension” was treated by surgically removing the splanchnic nerves [105,106]. The successful surgical interventions developed by Keith Grimson and Reginald Smithwick facilitated the development of chemical sympathectomy agents. However, the major backlashes of earlier surgical approaches were that it was invasive, time-consuming, and unsafe [107,108]. Over the past few decades, a return to surgical procedures has received much attention with the development of a brief (typically performed in 40 minutes) and less deleterious catheter-based renal denervation technique. The procedure only requires the insertion of a catheter into the femoral artery at the groin and advancing it to the renal artery. Radio frequencies are applied at the renal artery to ablate afferent and efferent sympathetic nerves connected to the kidneys [104,109]. 

In a proof-of-concept study, the SYMPLICITY HTN-1 study, Krum et. al [110] found that catheter-based renal denervation causes substantial and sustained BP reduction. However, they did not deem their findings decisive for managing resistant hypertension and called for more trials [111]. In a follow-up trial, SYMPLICITY HTN-2, similar outcomes were observed. Although both SYMPLICITY HTN-1 and HTN-2 trials had positive outcomes, they lacked sham controls, making the observations inconclusive [111,112,113]. To correctly affirm the suitability of renal denervation, well-designed randomized clinical trials are essential. In the third study with sham control (SYMPLICITY HTN-3), no significant difference was observed between the sham and treated groups. However, procedural insufficiencies such as fewer desirable ablations in the SYMPLICITY HTN-3 trial might be responsible for the unsuccessful findings. The failed attempt of the SYMPLICITY HTN-3 trial has left the fate of what appeared to be a promising treatment with unanswered questions. 

Regardless, the idea of this technique being relatively safer has caused it to receive much attention with a significant number of clinical trials been performed. Table 2 provides a list of some of the registered clinical trials involving renal denervation, their status, and the measured outcomes of their study.

### 5.4. Baroreceptor Reflex Activation

In 2019, the US Food and Drugs Administration approved the Barostim Neo^®^ System to improve symptoms in patients with heart failure [114]. The Barostim Neo system comprises an implantable pulse generator connected to a lead generator that is attached to the carotid artery in the neck. The device is programmed to deliver electrical impulses called baroreceptors which sense blood flow rate and relay them to the brain. The brain relying on this input transmits signals that regulate the heart and blood vessels to ensure optimum BP [114,115]. Although approved for managing heart failure, the technique employed in the device has previously been proposed for managing resistant hypertension since its first description over half a century ago [116,117]. Despite this promising feature, baroreceptor reflex activation has its pitfalls. Firstly, baroreceptor denervation significantly increases the short-term lability of arterial pressure but does not reduce arterial hypertension chronically. Also, in a phase III trial, the prototype device was associated with questionable efficacy and induced facial nerve palsy [104]. Fortunately, the production of a miniaturized second-generation pacing electrode allows the possibility of avoiding the significant side effect of facial nerve palsy [116].

### 5.5. Other Unconventional Strategies- Drugs Proposed and Drugs under Investigation

Recent studies show nitric oxide biosynthesis is impaired in hypertension, and its acute inhibition further exaggerates hypertension. Incidentally, nitric oxide levels are known to influence SNA (sympathoexcitation at a low level and inhibition at a high level). Although the mechanism behind this pathophysiology is not fully understood, stimulating nitric oxide synthesis has been proposed as a possible target for the treatment of NH. [104,118,119,120,121]. Although a conclusion of this potential therapy is not yet presented, a study confirmed that inhaled nitric oxide treatment could improve systemic oxygenation in infants with persistent pulmonary hypertension [122], further strengthening this claim.

Another unconventional therapy proposed is statins, a class of drugs more frequently prescribed for their potent cholesterol-lowering properties. Additionally, statins are known to stimulate nitric oxide production and possess anti-inflammatory and antioxidant properties [123,124,125,126]. In animal models of heart failure, statins were identified to lower renal SNA and cause marked improvements in heart rate variability indices of cardiac autonomic balance [104,123,127,128]. Although there is not enough data to claim statins’ suitability for hypertension, their potential to impact SNA requires further investigation.

Inflammatory molecules generated by vascular endothelial cells and leukocytes can infiltrate the brain parenchyma via disrupting the BBB. When these inflammatory chemicals interact with microglia, they release more ROS and chemokines/cytokines, which can have an immediate impact on neuronal function. As a result, the role of inflammation and oxidative stress in NH pathophysiology is another area of active research. Further, substantial evidence suggests that diets rich in antioxidants cause significant reductions in blood pressure [129]. Thus, food components such as oatmeal which are rich in antioxidants and can regulate metabolic syndrome, hold potential as a dietary approach to managing NH [130,131,132,133].

Also, neurosteroid allopregnanolone is identified as a potential treatment for NH. Allopregnanolone facilitates high-affinity extra-synaptic γ-aminobutyric acid A receptors through allosteric modulation and transcriptional upregulation.

## 6. The Problem of NH Management: The Barriers of the Central Nervous System

The CNS is protected from blood-borne substances by several interfaces, including the BBB, the blood–cerebrospinal fluid barrier, the cerebrospinal fluid–brain barrier, the blood–retinal barrier and the blood–spinal cord barrier. Although these barriers are intended to protect the CNS from extraneous material, they also prevent the permeation of useful therapeutics into the CNS [134,135]. Amongst the barriers, the major limiting factor to CNS permeation is the BBB.

A well-functional BBB controls homeostasis and ensures less trafficking of nonessential materials into the brain. Conversely, a dysfunctional BBB fails to control the flux of foreign substrates into the brain. Naturally, the integrity of the BBB is expected to decline with age; however, the BBB integrity is often compromised under certain pathological conditions. In NH, the increase in cerebral Ang II is augmented by the disrupted BBB, which not only permits inflammatory cell invasion but also facilitates the flux of peripheral Ang II [136].

The success of sympathectomy shows that proper control of CNS functions is key to the management of NH. Therefore, it is obvious that successful treatment of NH will only be achieved if the drugs can control the central effectors of BP along with peripheral effectors. Unfortunately, reaching the brain with the therapeutic concentration of drugs is a significant challenge in managing neurogenic disorders, and NH is no different. Despite multiple reports about a disrupted BBB in the pathogenesis of NH and hypertension as a whole [137], this situation still does not favor the flux of therapeutics into the brain. According to a meta-analysis by Ho et al., among ARBs, only telmisartan and candesartan are capable of crossing BBB [138] though at subtherapeutic levels.

Several studies prove that if physiological modifying agents can reach the cardioregulatory regions in the brain at therapeutic concentrations, NH can be adequately controlled or even prevented [139,140,141]. However, the major challenge for modern science is to find ways to deliver these potential therapeutics into the brain. Therefore, brain-targeted formulations that can cross the BBB and deliver medication at a therapeutic level are crucial for successful NH therapy.

## 7. Nanotherapeutics: A Potential Solution

Failure to achieve BP control in patients with NH suggests the need for treatment directed to the root cause rather than symptoms [7]. The advent of nanotherapeutics has dramatically accelerated the path to finding a cure for several healthcare problems [142,143]. Naturally occurring or fully engineered nanoparticles have become helpful in solving many medical emergencies [144,145]. In a simple sense, nanotherapeutics refers to formulations with sizes ranging from 1–1000 nm and are applicable for mitigating and treating medical conditions [143,146]. Nanotherapeutics have seen great success due to their numerous advantages, including small size, high surface-to-volume ratio, and tunable physiochemical properties, which allow them to mimic biological molecules [143,147].

**Figure 3:**
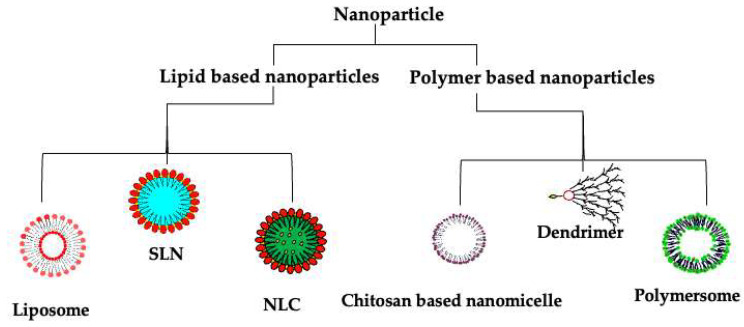
Current nanoparticles explored for neurogenic hypertension management. SLN; solid lipid nanoparticles, NLC; nanostructured lipid carriers.

Further, nanotherapeutics can be readily surface-functionalized to enable organ, tissue, or cell specificity. Since their introduction into medicinal sciences, several nanotherapeutics have gained approval for diagnosing, treating, and managing diverse medical conditions. In addition, several other nanotherapeutics are under investigation for use in disease management, and some have found use in cardiovascular disorders [148,149]. Thus, the scope of nanotherapeutics is broad, and there is still much to be discovered. Despite the great success of nanotherapeutics in managing conditions such as cancers, little application has been seen in NH management. The FDA has approved over 50 nanodrugs for diverse disease management, with many more currently under different phases of clinical trials, and this list continues to grow daily [150]. In the following sections, we will discuss some nanotherapeutics that have been used and investigated for the management of NH. We focus on nanocarriers currently studied in relation to antihypertensive therapy: lipid- and polymer-based nanotherapeutics, their use, and the challenges that restrict their possible success. These formulations are illustrated in Figure 3.

### 7.1. Lipid-Based Nanotherapeutics

Lipid-based nanocarriers have become popular in drug delivery due to their biocompatibility, low toxicity, and ease of functionalization to accomplish the desired goal, such as targeting [142,151]. The tendency for lipids to self-aggregate has made them especially useful as drug/gene carriers since they can easily entrap molecules within their core [152]. Commonly used lipids in formulations include phospholipids and fatty acids. While some lipids such as phospholipids and glycolipids are more suited for making vesicles, other lipids such as fatty acids and steroids are more helpful in achieving specific goals such as targeting and formulation rigidity. Lipid-based carriers include liposomes, solid lipid nanoparticles (SLNs), and nanostructured lipid carriers (NLCs) [152,153]. Recently, lipid-based carriers have become popular again after their staggering use in developing vaccines against COVID-19 [154].

#### 7.1.1. Liposomes

Liposomes are the earliest nanoparticle-based drug delivery systems to have been conceptualized and successfully implemented clinically for transporting hydrophobic and hydrophilic substances and genetic materials [155,156]. Liposomes are 20 to 1000-nm-sized spherical vesicles formulated using various phospholipids [157,158]. The ability to control liposome size, surface characteristics, and release profile makes them very versatile for use in the delivery of drugs and genes [159,160]. For instance, PEGylated liposomes can evade the reticuloendothelial systems allowing for improved circulation times [161]. In addition, liposomes can be functionalized with various ligands for targeting specific cells or tissues.

Since the registry of the first liposomal formulation, Ambisome, several other liposomal formulations have been developed for numerous applications [162]. Amongst the nanotherapeutics, liposomes are the most extensively studied in relation to hypertension. In a study, neutral unilamellar liposomes were used to carry indium-Ill and nitrilotriacetic acid into the arterial walls of aortic coarctation-induced hypertensive New Zealand White rabbits [163]. In this study, the uptake of these active agents was markedly increased in the arterial walls, suggesting the usefulness of liposomal formulations in managing hypertension. Similarly, unilateral microinjections of Ang-(1-7) liposomes into the RVLM altered the circadian mean arterial pressure and heart rate variations. In an isolated study, intravenously administered cationic liposomes encasing antisense oligodeoxynucleotide of Giα-2 and Giα-3 were found to attenuate the development of hypertension in SHR [164]. The blockade of Giα proteins effectively attenuated Ang II-induced increase in BP. In a similar study, liposome-encapsulated angiotensinogen antisense was administered to SHR to investigate the possible BP reduction through peripheral Ang II antagonism. Rats receiving liposome-encapsulated antisense showed significantly lower peripheral angiotensinogen and Ang II levels than control groups [165].

Liposomes have also been used to investigate the role of superoxide in Ang II-induced and catecholamine-induced hypertension. Liposome-encapsulated superoxide formulation was administered following Ang II or norepinephrine-mediated hypertension development. In this study, BP was effectively reduced using liposomal superoxide treatment [166]. Although the recent success of brain-targeted liposomes provides an avenue for their applications in many brain disorders, the use of liposomes in NH management has not received much attention [22,26,155,160,167]. In addition, the effect of liposomes on BP is still debated among some scientists. Szebeni et al. investigated the hemodynamic changes associated with administering multilamellar liposomes with or without encapsulated hemoglobin in pigs. They observed a dose-dependent rise in pulmonary arterial pressure and heart rate by the fifth minute, which eventually subsided by the twentieth minute upon injection of hemoglobin-free liposomes [168].

#### 7.1.2. Solid Lipid Nanoparticles (SLNs)

SLNs are submicron-sized lipid emulsions wherein the liquid lipid (oil) is substituted by a solid lipid. SLNs are spherical lipid structures like liposomes, except they are more physically stable with a complex internal architecture [146]. A significant advantage of SLNs is that they can remain solid at room and body temperature, thus can restrict drug mobility and consequently improve stability and control the timing and release rate of their cargo. [25,159,169]. This technology can prove helpful, especially for managing NH to achieve a controlled release of antihypertensive therapeutics in the brain [170]. Although SLNs can load large quantities of cargo when more complex lipids are used, their loading capacity is limited by the drug solubility within the lipid melt, lipid matrix structure, and physical state [170]. Despite this unpredictable behavior, the structure of conventional SLNs can be stabilized by incorporating polymers and surfactants [169,171]. Due to their favorable cell membrane permeabilities, SLNs are proposed as an effective way to cross the BBB, thus a potentially effective tool for use in NH management [169,172,173]. Although the presence of surfactants promotes brain uptake, SLNs can also be functionalized with targeting molecules to improve their performance [24,174].

Regarding hypertension, only a few studies have been performed using SLNs. For instance, the SLNs formulation of olmersartan medoxomil was found to be stable with better in vitro drug release and bioavailability following oral administration in rats [175]. In a similar but isolated study, a 2.32-fold improvement in the oral bioavailability of olmersartan was observed when administered as an SLN formulation compared to the free drug.

SLNs have also been used to improve the oral bioavailability of candesartan cilexetil [176,177,178]. In a separate study, the oral bioavailability and pharmacodynamic effect of candesartan cilexetil and nisoldipine were improved by formulating them into SLNs [179]. Similarly, SLNs increased the oral bioavailability of the calcium channel blockers isradipine and cilnidipine [180,181].

#### 7.1.3. Nanostructured Lipid Carriers (NLCs)

NLCs are often considered the second generation of lipid nanoparticles since they were developed particularly to overcome the drawbacks of using SLNs, including degradation and instability [182]. Similar to SLNs, constituents of NLCs determine their physicochemical properties and final product effectiveness; however, NLCs are made from both solid and liquid lipids [183]. Generally, NLCs release their entrapped cargos through diffusion and lipid degradation but mostly follow a biphasic pattern consisting of a prolonged release that follows an initial burst release [153]. Because they allow high drug entrapment and loading capacities for lipophilic drugs, NLCs are more suitable for entrapping drugs with high log *p* values [184]. However, NLCs are highly useful for transporting hydrophilic and hydrophobic substances across the BBB [185].

Alam et al. [186] formulated isradipine-loaded NLCs to improve the oral bioavailability of isradipine. The optimized NLCs with and without cyclohexamine (lymphatic transport inhibitor) exhibited 1.9 and 4.2 times increase in oral bioavailability, respectively. In another study, both medium and long-chain triglycerides were used to develop NLCs of perindopril [184]. It was observed that drug solubility was highest in the NLCs formulated with the solid lipid Emulcire 61. In a similar study, the oral bioavailability of olmesartan medoximil was improved by formulating into NLCs [187]. Kataria et al. [188] also developed and optimized NLCs to improve lacidipine’s oral bioavailability and antihypertensive activity. The NLC formulations exhibited a 3.45-fold higher relative bioavailability than the dispersed lacidipine. Although NLCs have found significant use in improving oral bioavailability, there is still room to investigate their impact on NH [189,190].

### 7.2. Polymer-Based Nanotherapeutics

Polymeric nanoparticles have recently become attractive platforms for drug and gene delivery. They can be loaded with active compounds either adsorbed to the surface or entrapped within the polymeric matrix [191]. Polymeric particles present several advantages, including biocompatibility, minimal toxicity, and ease of surface functionalization with targeting ligands [192]. The use of polymer-based nanoparticles for antihypertensive studies has mostly been centered on oral medications. Here we briefly discuss some polymer-based carriers and a few instances where they have featured in hypertension-related studies.

#### 7.2.1. Polymersomes

Polymersomes are made up of amphiphilic block copolymers with the ability of self-assembling to form hollow spheres of a bilayer membrane with an aqueous interior [193]. Unlike liposomes, polymersomes have higher stability and longer retention of their cargo. The amphiphilic copolymer ratios often determine physicochemical properties, surface activities, and characteristic behaviour in biological systems, such as stimuli responsiveness [148,194]. Not much work has been performed using polymersome-based antihypertensives. In a study, di-block mPEG-PCL copolymers were used to formulate polymersomes for the controlled delivery of enalapril [195].

#### 7.2.2. Polymeric Nanoparticles

Chitosan-based polymeric nanoparticles have been extensively explored among the different polymeric nanomicelles because of their biodegradable, biocompatible, and nontoxic nature [196]. Also, chitosan can be readily functionalized to achieve targeted drug delivery [197]. In a study, lecithin/chitosan nanoparticles entrapping ACE inhibitor ramipril produced a 1.6-fold decrease in systolic BP of salt-induced hypertensive rats compared to the free drug [198]. Also, Sharma et al. [199] prepared nebivolol-loaded chitosan nanomicelles to improve the oral bioavailability of the drug. According to their studies, chitosan nanoparticles showed a sustained drug release profile, with 71.24% of the entrapped drug released over 72 h.

Chitosan nanoparticles have also been used to deliver antihypertensive biopeptides. Auwal et al. [200] used ionotropic gelation fabricated nanoparticles to deliver stone fish-derived ACE-inhibitory biopeptides. They observed a significant dose-dependent BP-lowering effect of the biopeptides compared to the unencapsulated peptides. Further, chitosan has been suggested to have biological activities, including ACE inhibition, thus proving helpful in managing hypertension [196,201].

In addition to chitosan polymers, polysaccharide-based nanocarriers may be useful in NH management [202,203,204]. Some polysaccharides, such as lactoferrin, have been previously explored as nanotherapeutics. Lactoferrin is a glycoprotein having a nano self-assembly property [205]. Lactoferrin assembly produces cage-like structures with the tendency to disassemble and recombine with the absence or presence of iron [205]. In addition to these properties, the overexpression of the receptor on BBB makes lactoferrin an ideal nanocarrier for brain-targeted therapeutics delivery [206,207]. Despite this promise, very little is known about the suitability of lactoferrin-based nanoparticles for NH management.

#### 7.2.3. Dendrimers

Dendrimers are synthetic macromolecular structures with repetitive branched layers surrounding a central core molecule. The ability to fully disassemble dendrimers resulting in the release of conjugated molecules makes them a suitable platform for drug delivery [208,209,210,211]. Dendrimers have mostly received interest in managing ocular hypertension [208,212,213]. Second-generation phosphorus-containing dendrimers were tested for ocular delivery of carteolol (an ocular antihypertensive drug used to treat glaucoma). These dendrimers were found to increase the quantity of carteolol that penetrated the eyes of rabbits compared to carteolol alone [212]. In a separate study, poly (amidoamine) PAMAM dendrimers were simultaneously loaded with ramipril and hydrochlorothiazide [214].

## 8. Considerations for Improving Outcomes with Nanotherapeutics

The difficulty in reaching the brain (or specific regions within it) with therapeutic molecules makes managing NH even more challenging. Till now, different invasive procedures have been attempted to reach specific areas of the brain responsible for BP regulation; however, this can be avoided using efficient nanoparticle delivery systems [59]. In the following paragraphs, we briefly comment on how nanoparticles can be modified and used to achieve desirable outcomes in NH.

### 8.1. Surface Functionalization of Nanotherapeutics

A better understanding of their physicochemical characteristics is essential to improve nanotherapeutics performance. Previously, one major backlash to nanotherapeutics was their inability to deliver therapeutics at the desired site. The improvements in nanotechnology have provided strategies to ensure that cargo ferried by nanotherapeutics is released at the desired sites. A typical approach is the surface functionalization of nanocarriers with molecules that favor a biased uptake of nanotherapeutics into certain specific cells/tissues. Since its inception, surface functionalization has gained traction as an appealing way to improve the brain permeability of drug molecules. In a study by Jose et al. [215], surface functionalization of PLGA nanoparticles with Polysorbate 80 facilitated crossing the blood–brain barrier. Their study reported a higher brain concentration of the neuroprotective agent Bacoside-A reaching 23.94 ± 1.74 μg/g tissue, which was over ten times the pure drug-solution concentration in the brain. In a separate study by Rodriguez et al. [22], surface functionalization of liposomes with transferrin and a cell-penetrating peptide was sufficient to improve the brain permeability of liposomes in mice after a single i.v. administration. They observed the sufficient transport of dual functionalized liposomes in an in vitro BBB model and mice. In both of these studies, improvement in drug accumulation in the brain was attributed to surface functionalization. Surface functionalization can also improve the pharmacokinetic profile of the nanocarrier [216,217]. The numerous successful demonstrations of surface modification prove its suitability in enhancing brain delivery of therapeutics. Given that specific regions within the brain either promote or inhibit NH, it is also crucial that nanotherapeutics be functionalized with just the proper ligand to ensure it is directed towards the right structures.

### 8.2. Bioresponsive Nanoparticles

Recently, bioresponsive nanomedicines are becoming a popular strategy to overcome the protective effect of BBB and the release of cargo molecules under specific biological conditions or trigger [144,218,219]. Physiological changes such as oxidative stress [220] and inflammation present in NH can thus be used as stimuli for developing novel therapeutic tools. Physiological conditions can also be used to control drug release [221]. In a study, dual-sensitive nanomicelles were designed to deliver antibodies into the brain. In the conclusion of this study, the authors proposed that their delivery system can deliver a wide range of proteins into the brain [222].

### 8.3. Route of Administration

In addition, nose-to-brain transport is a promising strategy for delivering therapeutics to the brain since it provides a non-invasive route that bypasses the BBB [223]. The major cardioregulatory regions of the brain are located within the hypothalamus and the brain stem, directly behind the nasal cavity [223,224]. The direct placement of these regions behind the nose suggests that an attempt to administer formulations through the intranasal route could hold promising prospects towards managing NH. Nose-to-brain targeted delivery has been explored in several instances; most of these instances take advantage of the olfactory and trigeminal nerves and neuronal transport towards the hypothalamus [225,226,227]. Since the trigeminal nerves lead towards the brain stem, it could be a suitable route to target structures such as the NTS, RVLM, and CVLM [223]. Our previous study demonstrated that functionalized chitosan nanomicelles could target the brain when administered through the nasal route [197]. Compared to the intravenous route, the intranasal administration leads to a significantly (*p* < 0.05) higher VGF expression within the brain using multifunctionalized polymeric micelles.

## 9. Conclusions and Future Perspectives

Persistent hypertension is a cause and significant risk factor for renal impairment and other cardiovascular diseases. Recent understanding proves the brain plays a critical role in maintaining elevated blood pressure in NH. However, the protective role of BBB prevents therapeutics from reaching the target site in the brain, making successful NH therapy extremely challenging. Therefore, developing effective brain-targeted drug delivery strategies is critical to treat NH successfully.

Nanoparticles provide a great platform to efficiently deliver therapeutic agents to the brain, suggesting they would be a better approach to solving the problem of NH. Nevertheless, none of these studies are focused on NH therapy. Thus, to affirm nanomedicine’s role in NH management, several studies will have to be performed using the NH animal model. Furthermore, developing a correlation between in vitro and in vivo data is essential for selecting efficient nanoformulation for preclinical and clinical studies. Particularly, the behavior of the nanomedicines under pathological conditions needs to be fully addressed. The lack of effective NH treatment is also attributed partly to the lack of proper understanding of the pathophysiology of NH. In conclusion, many more studies will have to be performed to develop nanotherapeutics with desirable properties for use in NH.

## Figures and Tables

**Table 1:** Current antihypertensive medications in clinical use.

Drug Class	Mechanism	Examples	Special Notes
ACE inhibitors	Block the action of ACE to prevent the conversion of Ang I to Ang II	Benazepril, captopril, enalapril, fosinopril, lisinopril, moexipril, perindopril, quinapril, ramipril, trandolapril	Used in combination with other therapeutic agents [99].
ARBs	Reduce the action of Ang II at its receptor by blocking it, causing vasodilation.	Azilsartan, eprosartan, losartan, irbesartan, olmesartan, valsartan, telmisartan, candesartan	Eprosartan possesses sympathoinhibitory [100].
Calcium channel blockers	Prevents calcium influx into heart and arterial cells by blocking the calcium channels.	Nifedipine, amlodipine, felodipine, nicardipine, isradipine, nisoldipine, diltiazem, verapamil	
Diuretics	Induce excretion of body salt and water by aiding in the movement of sodium into the urine.	Chlorothiazide,^#^ indapamide,^#^ metolazone,^#^ chlorthalidone,^#^ hydrochlorothiazide,^#^ bumetanide,^*^ ethacrynic acid,^*^ furosemide,* torsemide,* spironolactone,^&^ amiloride,^&^ eplerenone,^&^ triamterene^&^	They can be used as monotherapy or in combination. Three types of diuretics are in clinical use: thiazide,^#^ loop,^*^ and potassium sparing^&^
Aldosterone antagonist	Block the action of aldosterone resulting in salt and water loss.	Spironolactone, eplerenone, finerenone	
α-blockers	Partially block alpha-adrenergic receptor activity.	Doxazosin, prazosin, terazosin	They are used in combination therapies. Improves urine flow in older men with prostate problems.
Vasodilators	Improve blood flow by relaxing blood vessels.	Hydralazine, minoxidil	
β-blockers	Block beta receptors	Atenolol, pindolol, metoprolol, propranolol, bisoprolol, timolol, labetalol, carvedilol, acebutolol	Not generally recommended as first-line drugs
Renin-inhibitors	Inhibit the enzyme renin from triggering a process that helps regulate BP.	Aliskiren	Have an additive effect when used with diuretics
Centrally acting antihypertensives (α-2 agonist)	Stimulate presynaptic alpha2-adrenergic receptors in the brain stem, which reduces SNA	Methyldopa, clonidine, guanfacine	

Symbols present on examples of diuretic represent their class, ("^#^”,”^*^” and “^&^” represent Thiazide Loop and Potassium sparing diuretics respectively

**Table 2:** Clinical trials involving surgical treatments for drug resistant hypertension.

Study Title	Status	Interventions	Primary Measured Outcome	Clinicaltrials.gov Identifier
Efficacy and safety of renal sympathetic denervation from the adventitia on resistant hypertension	Unknown	Renal denervation via radiofrequency ablation instruments	Change in 24-hour average systolic BP	NCT03758196
A pragmatic randomized clinical evaluation of renal denervation for treatment resistant hypertension	Terminated	Renal denervation	Average systolic 24-hour ambulatory BP	NCT01895140
Renablate feasibility study cs156 (EC12-02) study of catheter based renal denervation to treat resistant hypertension	Completed	Renal denervation using celsius^®^ thermocool^®^	Incidence of major cardiovascular and/or renal adverse events related to the renal denervation procedure that occurred within 30 days post-procedure.	NCT01756300
The effect of baton bp and sympathetic function in resistant hypertension (the Nordic BAT study) (BAT)	Active	Baroreflex activation therapy	Change in systolic ambulatory BP in response to bat therapy	NCT02572024
Renal denervation in treatment resistant hypertension (ReSET-2)	Terminated	Ablation of the renal arteries	Change from baseline in daytime systolic BP (24-hour ambulatory bp measurement	NCT01762488
Treatment of resistant hypertension using renal denervation in china (REDUCE-HTN-CN)	Terminated	Percutaneous renal denervation with the vessix™ renal denervation system	The mean reduction of systolic bp measured using office-based BP assessment	NCT02027012
Treatment of resistant hypertension using a radiofrequency percutaneous transluminal angioplasty catheter (REDUCE-HTN)	Completed	Renal denervation	Change in systolic and diastolic bp at six (6) months as measured using office-based BP assessment	NCT01541865
Rapid renal sympathetic denervation for resistant hypertension ii (RAPID II)	Withdrawn	Renal denervation using one-shot™ renal denervation system)	Major adverse event (MAE) rate through 30 days post-randomization	NCT01939392
Renal sympathetic denervation in patients with drug-resistant hypertension and symptomatic atrial fibrillation	Unknown	Renal sympathetic denervation	Change in atrial fibrillation burden	NCT01713270
Feasibility study of renal denervation for the treatment of resistant hypertension	Unknown	Ultrasound-based renal denervation	Major adverse events	NCT01865591
Renal denervation for management of drug-resistant hypertension (INSPiRED)	Completed	Renal denervation	Change in systolic BP from baseline to 6 months on 24-h ambulatory measurement	NCT01505010
Randomized controlled trial of renal denervation for resistant hypertension	Unknown	Renal denervation using radiofrequency ablation catheter with drug treatment: amlodipine, losartan potassium and hydrochlorothiazide	Change in average 24-hour systolic BP using ambulatory bp monitoring from baseline	NCT02900729
Renal denervation in treatment resistant hypertension	Completed	Renal denervation using symplicity catheter system	Change in office BP from baseline to 6 months post-renal denervation	NCT01687725
Effects of renal sympathetic denervation on the cardiac and renal functions in patients with drug-resistant hypertension through mri evaluation (RDN)	Unknown	Renal denervation (enligHTN™) with the enligHTN™ renal denervation system.	Cardiac function (evaluated using MRI)	NCT02164435
